# The effectiveness of acupuncture in the treatment of Tourette syndrome in Chinese children: a systematic review and meta-analysis

**DOI:** 10.3389/fpubh.2025.1677592

**Published:** 2025-10-02

**Authors:** Qian-Qian Zhou, Zi-Chen Li, Zhuo-Ya Hu, Juan Tang, Peng Tang, Qi-Rui Wu, Zhong-Qi Deng, Wen-Bin Ma, Lei Lan

**Affiliations:** ^1^Acupuncture and Tuina College, Chengdu University of Traditional Chinese Medicine, Chengdu, Sichuan, China; ^2^School of Health and Rehabilitation, Chengdu University of Traditional Chinese Medicine, Chengdu, Sichuan, China

**Keywords:** acupuncture, Tourette syndrome, children, systematic review, meta-analysis

## Abstract

**Objective:**

As a prominent complementary and alternative therapy, acupuncture is widely used to treat Tourette syndrome in children. This review aims to evaluate its clinical efficacy and provide evidence-based support for acupuncture in pediatric Tourette syndrome.

**Methods:**

We systematically searched six databases: China National Knowledge Infrastructure, Wanfang Database, VIP Information Chinese Journal Service Platform, PubMed, Cochrane Central Register of Controlled Trials, and Embase, from their inception to 10 April 2025. Randomized controlled trials comparing acupuncture alone versus medication, or acupuncture plus other treatments versus other treatments alone, for children tic disorder were included.

**Results:**

Thirty-two studies were included, with 2,201 participants. Acupuncture may be more effective in improving motor tics symptoms than dopamine agonist [WMD −3.04, 95% CI (−3.77, −2.31), RD 0.38 (0.29, 0.46)], slightly improving vocal tics [WMD −2.39, 95% CI (−3.51, −1.26), RD 0.21 (0.10, 0.35)] and overall condition [WMD −5.56, 95% CI (−7.28, −3.83), RD 0.05 (0.02, 0.09)], but having little difference in functional impairment [WMD −2.27, 95% CI (−3.58, −0.96), RD 0.14 (0.09, 0.20)]. Acupuncture may be more effective than blank treatment on basis of other therapies in improving motor tics [WMD −2.51, 95% CI (−3.54, −1.49), RD 0.31 (0.19, 0.41)] and vocal tics [WMD −2.56, 95% CI (−3.66, −1.45), RD 0.28 (0.15, 0.40)], but slightly improving functional impairment [WMD −2.91, 95% CI (−4.64, −1.19), RD 0.13 (0.05, 0.23)] and overall symptom severity [WMD −5.57, 95% CI (−7.47, −3.68), RD 0.11 (0.06, 0.17)].

**Conclusion:**

Chinese children with Tourette syndrome using acupuncture may experience more improvement in motor tics symptoms than those using dopamine agonist. Acupuncture combined with other therapies may bring Chinese children with Tourette syndrome symptom relief in motor tics and vocal tics more than those alone. All results are supported by low-quality evidence.

**Systematic review registration:**

https://www.crd.york.ac.uk/PROSPERO/view/CRD42023444312, identifier CRD42023444312.

## 1 Introduction

Tourette’s syndrome (TS) is a prevalent chronic neuropsychiatric disorder representing a distinct chronic subtype of tic disorders: onset occurs before age 18, with a duration exceeding 1 year; it involves both motor and vocal tics, which may occur asynchronously. This distinguishes it from chronic tic disorders presenting solely with one tic type and transient tic disorders lasting less than 1 year. Patients typically present with semivoluntary muscle twitching in the head, face, shoulders, neck, and limbs, alongside abnormal vocalizations. These symptoms are frequently accompanied by psychological comorbidities such as attention deficit hyperactivity disorder and obsessive-compulsive disorder (1). The causes and mechanisms of the disease remain unclear, and treatment primarily involves symptomatic drug therapy (2). Long-term medication use often leads to adverse neurological reactions, such as blurred vision, drowsiness, fatigue, nausea, and vomiting, making it difficult for children to comply with long-term medication regimens, resulting in suboptimal treatment outcomes (3). Acupuncture is a non-pharmacological therapy for neurological disorders. Research indicates that acupuncture has demonstrated substantial efficacy in treating such conditions, including central nervous system disorders such as stroke and migraine, and peripheral nervous system disorders such as Bell’s palsy and trigeminal neuralgia. With its minimal adverse reactions, acupuncture is gaining increasing acceptance among practitioners and patients alike (4, 5). This study aims to evaluate the clinical efficacy of acupuncture in treating children with TS and provide evidence-based medical for acupuncture treatment of childhood Tourette syndrome.

## 2 Methods

### 2.1 Literature search

We followed the Preferred Reporting Items for Systematic Reviews and Meta-Analyses (PRISMA) guidelines (6) to report our systematic review and registered it in PROSPERO (registration number: CRD42023444312). The search strategy is as follows: (1) Database Search: China National Knowledge Infrastructure, Wanfang Data Online Knowledge Service Platform, VIP Information Chinese Journal Service Platform, PubMed, Cochrane Central Register of Controlled Trials, and Embase Database. (2) Manual Search: (1) China Clinical Trials Registry (search terms: Tourette syndrome AND acupuncture); (2) Included literature from published systematic reviews and their reference lists. The search was conducted up to 10 April 2025, with no language restrictions. (Database search terms and search results are listed in Supplementary Table 1).

### 2.2 Literature screen and data extraction

After removing duplicate documents using Endnote 21 software, four researchers (QQZ, ZCL, ZYH, JT) independently read the titles, abstracts, and full texts of all documents to determine the final documents to be included. In case of disagreement, the issue was resolved through discussion or by the third party (PT, LL). Data extraction was conducted independently by four researchers (QQZ, ZCL, ZYH, JT) in a blinded fashion. The extracted data included: literature characteristics (title, authors, publication year, age, disease duration, sample size, intervention and control measures), methodological information (randomization method, allocation concealment, use of blinding, loss to follow-up), and outcome measures (tics, vocal tics, functional impairment, adverse reactions). After extraction, we applied the following inclusion and exclusion criteria.

Inclusion criteria: (1) patients diagnosed with TS according to the diagnostic criteria of the Diagnostic and Statistical Manual of Mental Disorders, Fifth Edition (DSM-5) (7), with a disease duration of over 1 year and no tic-free periods exceeding 2 months, aged <18 years,(2) acupuncture vs. western medicine, acupuncture vs. blank control, with a follow-up ≥4 weeks, (3) randomized controlled trial (RCT), (4) primary outcomes involved tic symptoms, coprolalia symptoms, and functional impairment, and secondary outcomes were adverse reactions.

Exclusion criteria: (1) Semi-randomized controlled trials; (2) Tics attributable to other neurological disorders.

### 2.3 Risk of bias assessment

The risk of bias in the included RCTs was assessed independently by two reviewers (QQZ, ZCL) using the Cochrane Risk of Bias 1.0 tool (8, 9). The following domains were rated: (1) random sequence generation; (2) allocation concealment; (3) blinding of participants and personnel; (4) blinding of outcome assessment; (5) incomplete outcome data; (6) selective reporting; and (7) other sources of bias.

### 2.4 Data analysis

Continuous variables were analyzed using the weighted mean difference (WMD) and corresponding confidence interval (95% CI); binary variables were analyzed using the relative risk (RR) and 95% CI. All meta-analyses were performed using a random-effects model in Stata 17 software. Heterogeneity was assessed using the I^2^ value and Q test, with an I^2^ value ≥ 50% indicating high heterogeneity. Subgroup analysis was conducted to identify sources of heterogeneity. To better interpret the clinical significance of effect sizes, we defined an effect size of ≥30% reduction from baseline as the minimal clinically important difference (MCID), and calculated the risk difference (RD) between groups to quantify between-group differences.

### 2.5 Certainty of evidence

We used the GRADE (Grading of Recommendations, Assessment, Development, and Evaluation) method to assess the certainty of evidence for each outcome (10). Evidence from RCTs started at high quality but can be downgraded to high, moderate, low, or very low quality based on factors such as risk of bias, consistency, directness, precision, and publication bias.

## 3 Results

### 3.1 Literature screening

**Figure 1:**
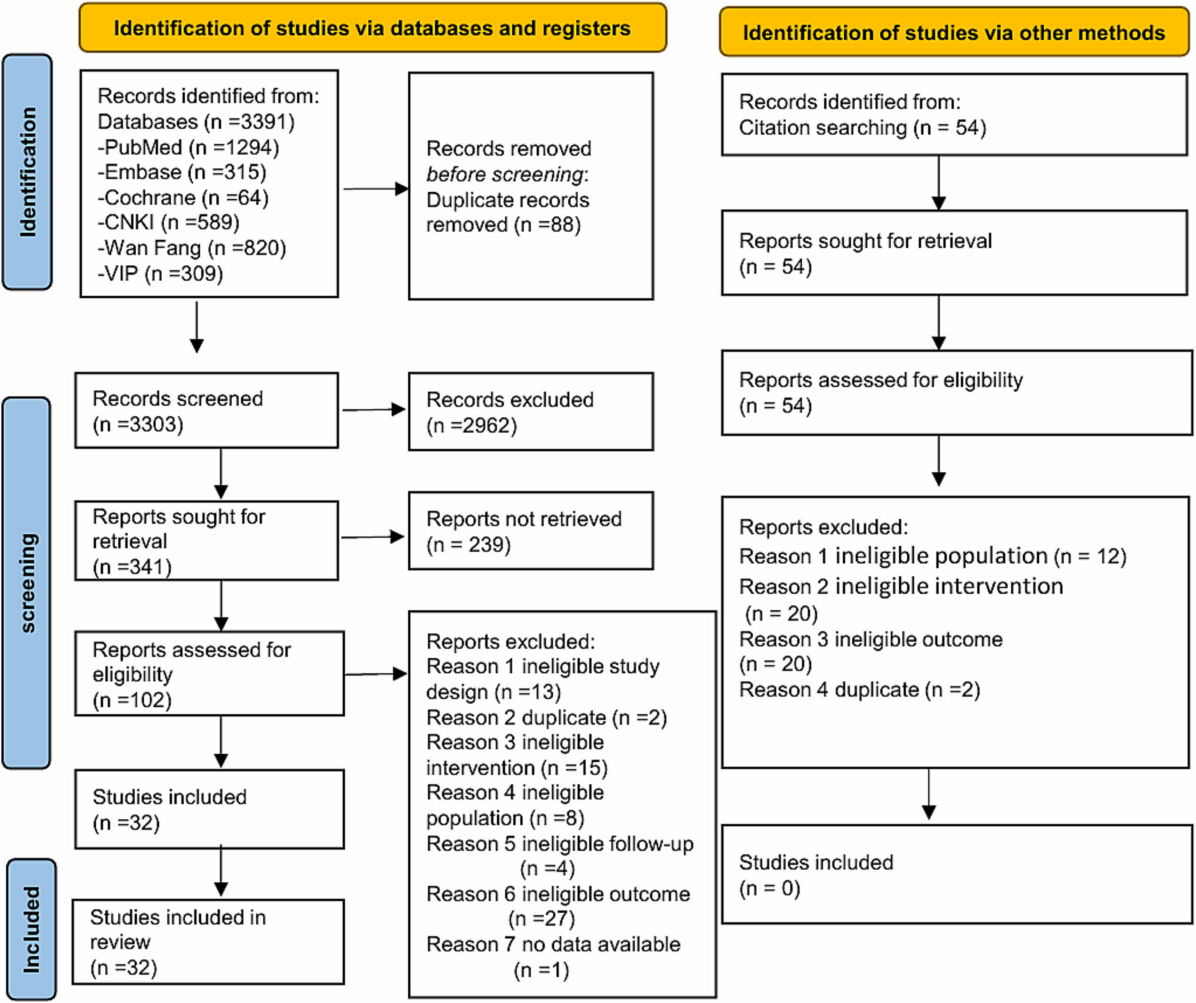
Literature screening process and results of acupuncture for children with TS.

Database search yielded 3,391 records. Manual search yielded 54 records from 11 published systematic reviews (11–21). Thirty-two studies were ultimately included (Figure 1).

### 3.2 Characteristics of included studies

Thirty-two studies (22–52) were included, involving 2,201 patients, with 28.17% being female. All included studies were conducted in China, and 10 studies (22, 25, 29, 34, 36, 42–44, 48, 51) received funding support. Among the 25 studies reporting participants’ specific ages, the average age of participants was 8.84 years, with the shortest intervention duration being 28 days and the longest being 120 days. This included 15 acupuncture vs. medication control studies (22–31, 33–36, 52), 11 acupuncture combined with medication vs. medication alone control studies (38, 39, 41, 43, 44, 46–50, 52, 53), 4 studies comparing acupuncture combined with Chinese herbal medicine vs. Chinese herbal medicine alone (32, 37, 45, 51), 1 study comparing acupuncture combined with ear acupuncture vs. ear acupuncture alone (42), and 1 study comparing acupuncture combined with cognitive behavioral therapy vs. cognitive behavioral therapy alone (40). Fourteen studies (22, 24, 28–30, 33–35, 43, 44, 46, 48, 49, 51) reported adverse reactions. The detailed information of the included studies can be seen in Table 1.

**Table 1:** Baseline characteristics of included studies.

Study ID	Country	Funding	Treatment (No.)	Control (No.)	Sample size	Female (%)	Age (years) Mean ± SD Median (P25, P75)	Treatment duration	Outcomes
Le W 2022	China	Government	Acupuncture 30	Tiapride 30	60	46.67%	10.4 ± 3.03 10.4 (8.3, 12.5)	4 M	④⑤
Li YC 2016	China	NR	Acupuncture 32	Inosine tablets & Vitamin B6 32	64	32.81%	8.25 ± 2.9 8.3 (6.2, 10.3)	1 M	④
Liu H 2016	China	NR	Acupuncture 40	Haloperidol 40	80	22.5%	9 ± 2.53 9 (7.3, 10.7)	2 M	④⑤
Liu L 2010	China	Government	Acupuncture 30	Haloperidol 30	60	31.67%	8.6 ± 3.2 8.6 (6.4, 10.8)	2 M	①②③④
Mu JP 2009	China	NR	Acupuncture & CBT 60	Haloperidol & CBT 60	120	44.17%	9.9 ± 3.11 9.9 (7.8, 12)	6 W	①②③④
Ni W 2016	China	NR	Acupuncture 29	Inosine tablets & Vitamin B6 28	57	40.35%	8.66 ± 3.21 8.7 (6.5, 10.8)	4 W	④
Song Y 2022	China	NR	Acupuncture 41	Tiapride 37	78	30.77%	8.2 ± 0.77 8.22 (7.7, 8.74)	3 M	④⑤
Sun YZ 2018	China	Government	Acupuncture 30	Haloperidol 30	60	21.67%	14.9 ± 6.05 14.92 (10.8, 19)	8 W	④⑤
Wang SJ1 2020	China	NR	Acupuncture 30	Tiapride 30	60	18.33%	8.9 ± 2.26 8.9 (7.3, 10.4)	4 W	①②③④⑤
Xu SF 2009	China	NO	Acupuncture 30	Risperidone 30	60	11.67%	9.5 ± 2.9 9.5 (7.5, 11.5)	3 M	①②③④
Yin ZQ 2024	China	NR	Acupuncture 40	Changma Xifeng Tablets 39	79	30.38%	7.71 ± 1.82 12 (7, 12)	8 W	①②④⑤
Zhang ML 2024	China	Government	Acupuncture 45	Tiapride 45	90	27.78%	7.5 ± 1.28 7.46 (6.59, 8.33)	8 W	④⑤
Hu CY 2022	China	NR	Acupuncture 24	Haloperidol 24	48	47.92%	8 ± 2.15 7.96 (6.65, 9.27)	8 W	①②⑤
Dong ZW 2022	China	Government	Acupuncture & CBT 24	Haloperidol & CBT 24	48	47.92%	8 ± 2.15 7.96 (6.65, 9.27)	8 W	①②③④
Jiang JS 2020	China	NR	Acupuncture & Chinese medicine 22	Chinese medicine 41	63	9.52%	7.7 ± 2.16 7.63 (6.48, 8.78)	50D	④
Huang N 2011	China	NR	Acupuncture & Chinese medicine 30	Chinese medicine 30	60	25%	8.2 ± 2.76 8.15 (6.47, 9.83)	3 M	④
Liu J 2006	China	NR	Acupuncture & Haloperidol 30	Haloperidol 30	60	21.67%	12.14 ± 3.23 12.14 (10.03, 14.25)	1 M	①②③④
Ma JJ 2023	China	NR	Acupuncture& Haloperidol 30	Haloperidol 30	60	28.33%	6.3 ± 1.29 6.34 (5.52, 7.16)	4 W	④
Qi YJ 2020	China	NR	Acupuncture & CBT 20	CBT 20	40	17.5%	9.5 ± 2.42 9.53 (8.14, 10.91)	3 M	④
Shu LH 2023	China	NR	Acupuncture & Haloperidol & CBT 38	Haloperidol & CBT 38	76	28.95%	8.8 ± 1.5 8.75 (7.84, 9.66)	3 M	①②④
Song SF 2023	China	Government	Acupuncture & auricular points 40	auricular points 40	80	31.25	9 ± 1.29 9.02 (8.16, 9.88)	3 M	①②③
Wang D1 2024	China	Government	Acupuncture & Haloperidol 46	Haloperidol 46	92	16.3%	6.8 ± 2.47 6.75 (5.57, 7.93)	4 W	①②⑤
Wang SJ2 2023	China	Government	Acupuncture & Tiapride 40	Tiapride 40	80	27.5%	7.35 ± 1.88 7.35 (6.27, 8.43)	4 W	④⑤
Wu HS 2020	China	NR	Acupuncture & Chinese medicine 33	Chinese medicine 33	66	30.30%	9 ± 2.76 9 (7.4, 10.6)	6 W	①②③④
Xia Y 2022	China	NR	Acupuncture & Tiapride 31	Tiapride 31	62	40.32%	8 ± 2.25 7.95 (6.62, 9.28)	2 M	①②④⑤
Yang LX 2007	China	NR	Acupuncture & Haloperidol & CBT 56	Haloperidol & CBT 30	86	13.95%	11.7 ± 3.07 11.7 (9.81, 13.59)	48D	④
Zhang Y 2019	China	NR	Acupuncture & Haloperidol 35	Haloperidol 35	70	28.57%	9.1 ± 1.78 9.1(7.81, 10.39)	8 W	①②
Zhou YF 2023	China	Government	Acupuncture & Clonidine 33	Clonidine 33	66	21.21%	8.5 ± 2.44 8.45(7.13, 9.77)	8 W	④⑤
Kong Y 2017	China	NR	Acupuncture & Haloperidol 20	Haloperidol 20	40	32.5%	9 ± 2.83 8.92(7.71, 10.13)	8 W	①②③④⑤
Wang D2 2018	China	NR	Acupuncture & Tiapride 54	Tiapride 51	105	19.08%	8.9 ± 3.27 8.69(7.15, 10.23)	1 M	①②④
Zhu BC 2020	China	Government	Acupuncture & Chinese medicine 36	Chinese medicine 35	71	21.13%	9.5 ± 3.2 9.53(7.92, 11.14)	12 W	①②③④⑤
Yu PB 2019	China	NR	Acupuncture 30	Tiapride 30	60	45%	8 ± 1.77 8(6.94, 9.06)	1 M	④

NR, not reported; D, days; W, weeks; M, months; CBT, Cognitive behavioral therapy; ①: motor tic scores of YGTSS total effective rate; ②: motor tic scores of YGTSS; ③: impairment scores of YGTSS; ④: total score of the YGTSS; ⑤: adverse events.

### 3.3 Risk of bias

All of the 32 included studies showed that 26 (81%) had adequate randomization sequence generation, 3 (9%) had adequate allocation concealment (22, 35, 36), and 3 (9%) had blinding of data collectors, data processors, and data analysts (22, 35, 36). Six studies reported patient attrition (27, 32, 37, 48, 51, 53), but the attrition rates did not exceed 20% in any case (Supplementary Table 3).

### 3.4 Acupuncture vs. medicine

#### 3.4.1 Motor tic symptoms

**Figure 2:**
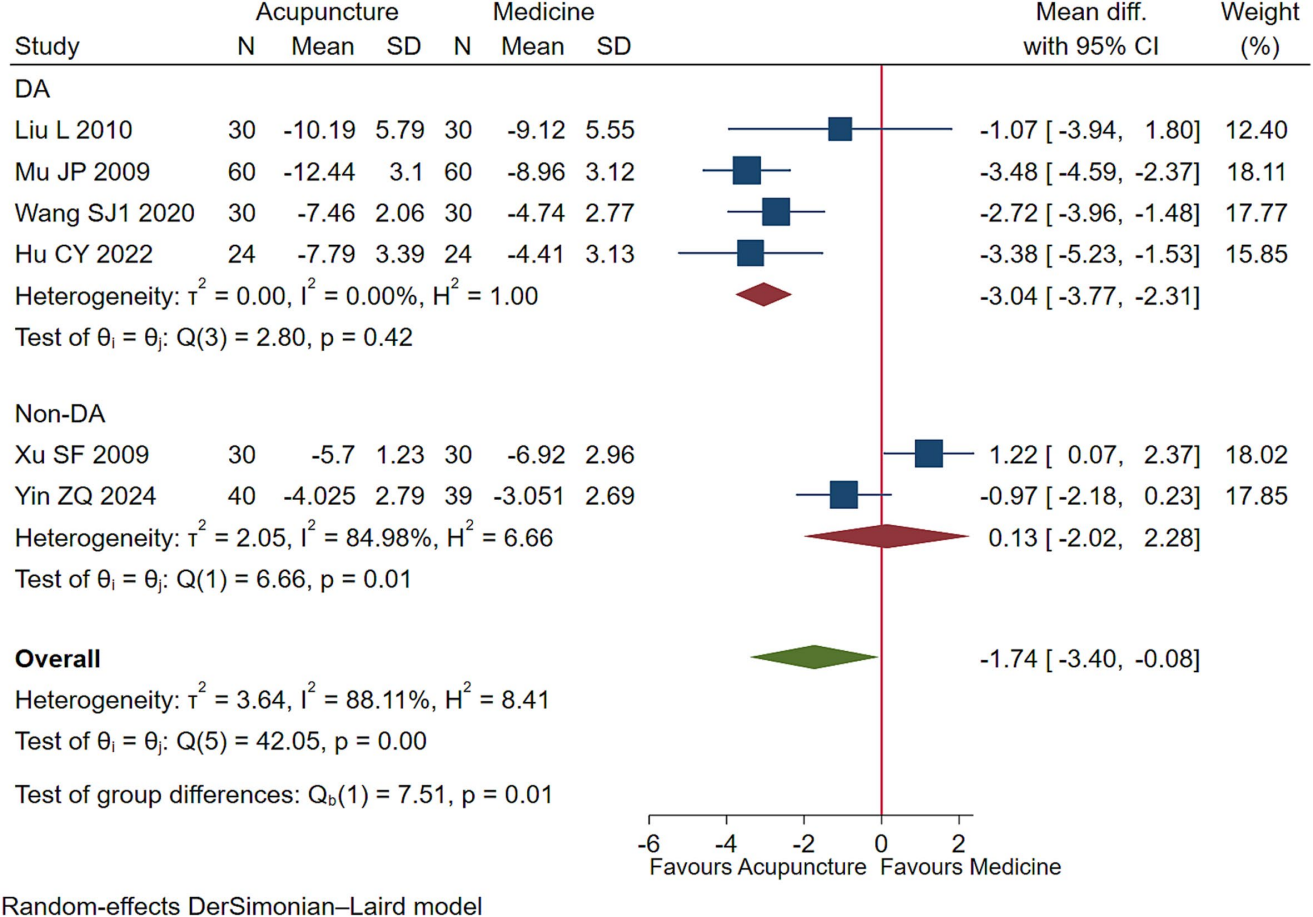
Acupuncture vs. medicine evaluation of efficacy of motor tics.

Low-quality evidence (4 studies, 288 participants) suggested that acupuncture may lessen motor tics symptoms in children with TS than DA [WMD −3.04, 95% CI (−3.77, −2.31), RD 0.38 (0.29, 0.46) (Figure 2; Table 2)] (25, 26, 30, 35).

**Table 2:** GRADE evidence quality rating of the clinical efficacy of acupuncture versus medicine therapy for children with TS.

Comparison	No. of trials (No. of patients)	Follow-up days	Risk of bias	Inconsistency	Indirectness	Imprecision	Publication bias	Treatment association (95% CI)		Overall quality of evidence
Acupuncture vs. Medicine	Motor Tic Score, 0–25, lower is better									
	4 (288)	28 to 60	Serious^a^	Not serious *I*^2^ = 0%	Not serious	Serious^b^	NA	WMD −3.04 (−3.77, −2.31)		Low^d^
								RD 0.38 (0.29, 0.46)		
								Acupuncture 67.4%	Medicine 29.1%	
	Vocal Tic Score, 0–25, lower is better									
	7 (475)	28 to 90	Serious^a^	Serious *I*^2^ = 81.46%	Not serious	Serious^b^	NA	WMD −2.39 (−3.51, −1.26)		Low
								RD 0.21 (0.10, 0.35)		
								Acupuncture 32.1%	Medicine 10.8%	
	Impairment Score, 0–50, lower is better									
	4 (300)	28 to 90	Serious^a^	Not serious I^2^ = 27.24%	Not serious	Serious^b^	NA	WMD −2.27 (−3.58, −0.96)		Low
								RD 0.05 (0.02, 0.09)		
								Acupuncture 10.3%	Medicine 5.3%	
	Global Severity Score, 0–100, lower is better									
	13 (993)	28 to 120	Serious^a^	Serious *I*^2^ = 77.41%	Not serious	Serious^c^	NA	WMD −5.56 (−7.28, −3.83)		Low
								RD 0.14 (0.09, 0.20)		
								Acupuncture 21.3%	Medicine 7.3%	
	Adverse reactions									
	7 (476)	28 to 120	Serious	Not serious *I*^2^ = 10.3%	Not serious	Serious^b^	NA	RR 0.34, 95%CI (0.24, 0.49)		Low^e^

NA, not available.

a, high risk bias in blinding and randomization; b, small sample size; c: we rated down two levels for imprecision when the ratio of the upper to the lower limits of the CI is more than three for risk ratio based on GRADE guidance; d, Subgroup analyses were conducted; e, A subgroup analysis was conducted after excluding one article to analyses the DA group.

#### 3.4.2 Vocal tic symptoms

**Figure 3:**
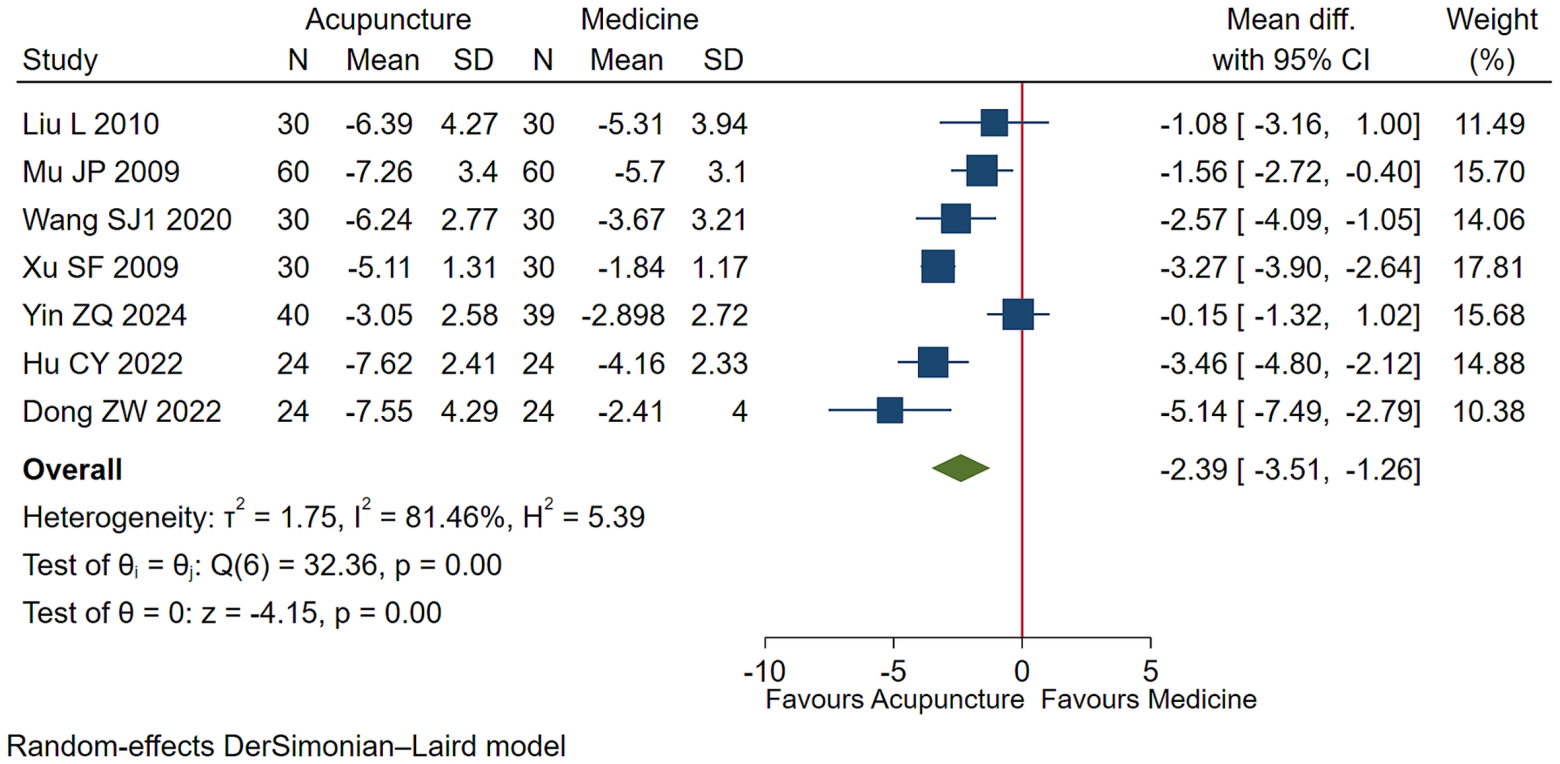
Acupuncture vs. medicine evaluation of efficacy of vocal tics.

Low-quality evidence (7 studies, 475 participants) suggested that acupuncture may slightly improve vocal tics symptoms in children with TS compared to conventional medication [WMD −2.39, 95% CI (−3.51, −1.26), RD 0.21 (0.10, 0.35) (Figure 3, Table 2)] (25, 26, 30, 31, 33, 35, 36).

#### 3.4.3 Functional impairment

**Figure 4:**
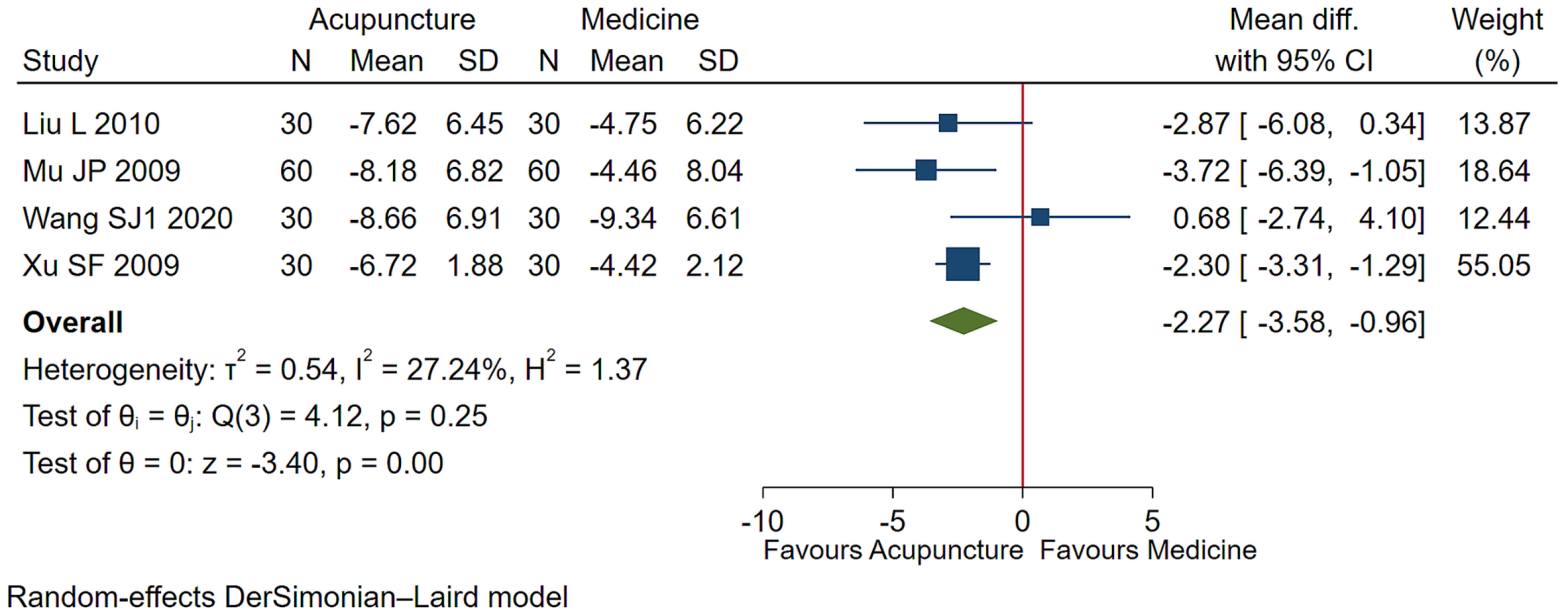
Acupuncture vs. medicine evaluation of efficacy of functional improvement.

Low-quality evidence (4 studies, 300 participants) suggested that acupuncture may has a negligible effect on functional impairment in children with TS [WMD −2.27, 95% CI (−3.58, −0.96), RD 0.05 (0.02, 0.09) (Figure 4; Table 2)] (25, 26, 30, 31).

#### 3.4.4 Overall symptom severity

**Figure 5:**
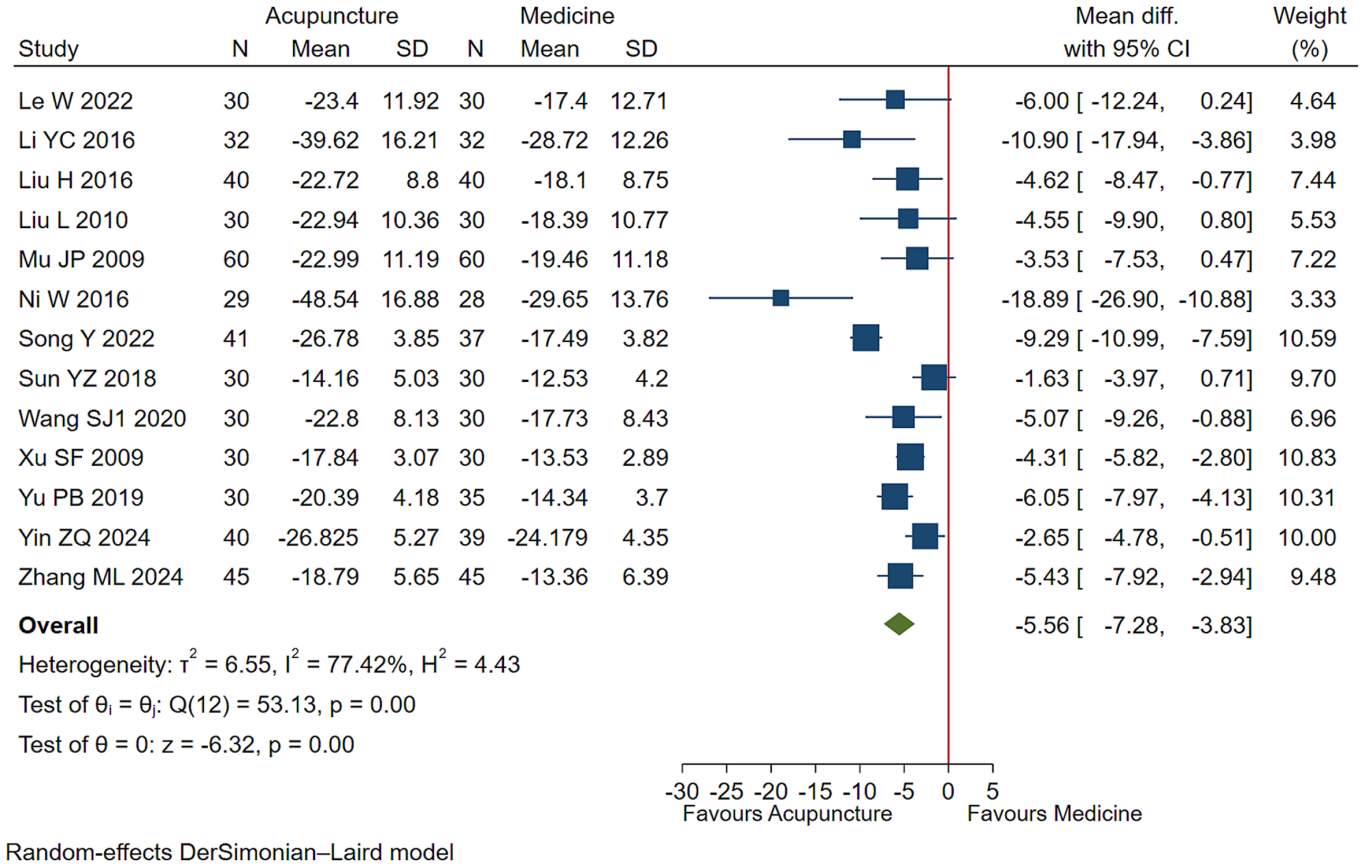
Acupuncture vs. medicine evaluation of efficacy of overall symptom efficacy evaluation.

Low-quality evidence (13 studies, 995 participants) suggested that acupuncture may slightly control overall symptom severity in children with TS [WMD −5.56, 95% CI (−7.28, −3.83), RD 0.14 (0.09, 0.20) (Figure 5; Table 2)] (22–31, 33, 34, 52).

#### 3.4.5 Adverse reactions

**Figure 6:**
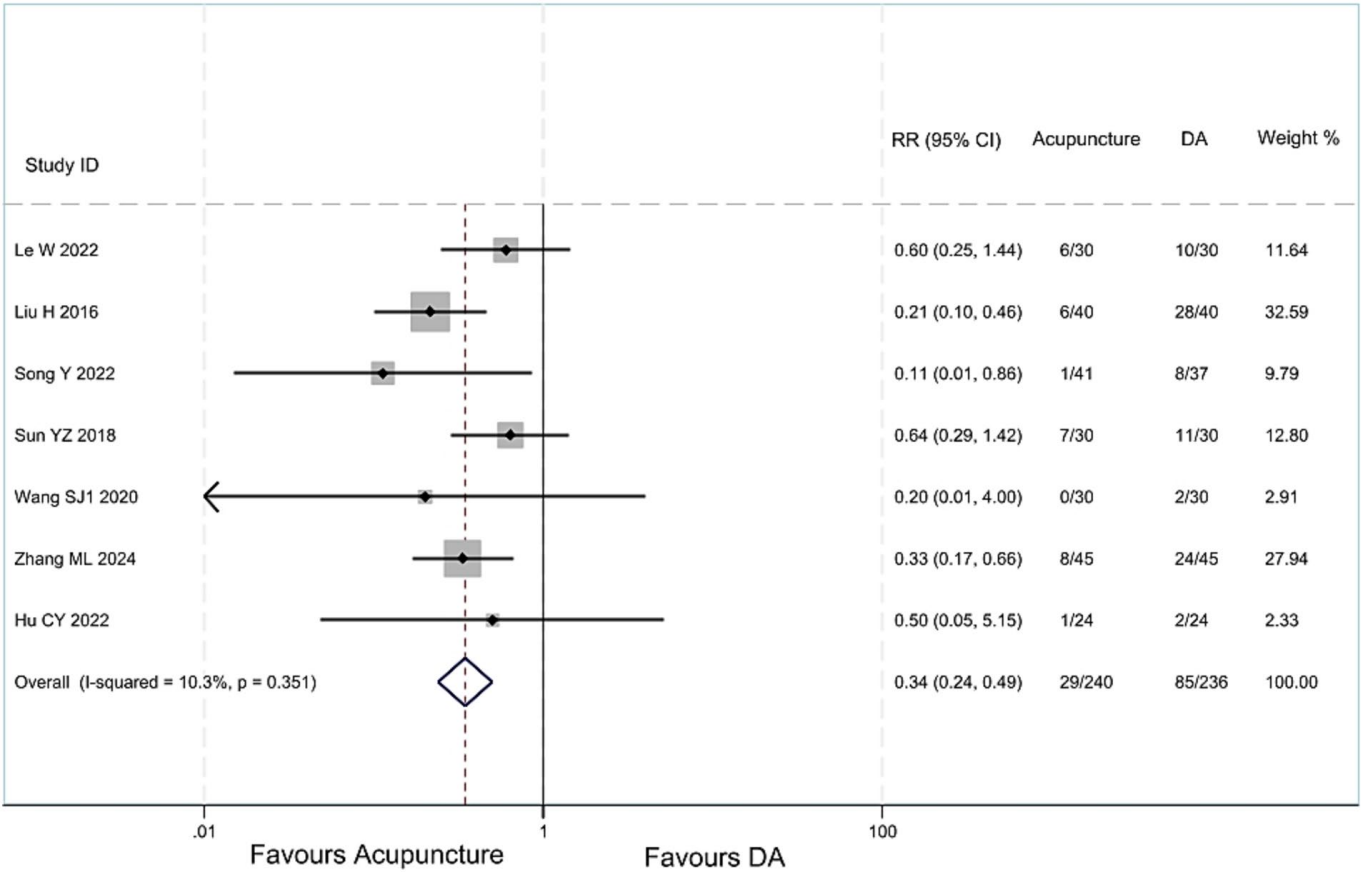
Acupuncture vs. DA group analysis of the incidence of adverse reactions.

Low-quality evidence (7 studies, 476 participants) suggested that acupuncture treatment for children with TS has less adverse reactions than DA, [RR 0.34, 95%CI (0.24, 0.49) (Figure 6; Table 2)] (22, 24, 28–30, 34, 35). One article (33) compared the acupuncture group with the drug group, and found no difference in the incidence of adverse reactions between the two groups.

### 3.5 Acupuncture vs. blank control on basis of usual care

#### 3.5.1 Motor tic symptoms

**Figure 7:**
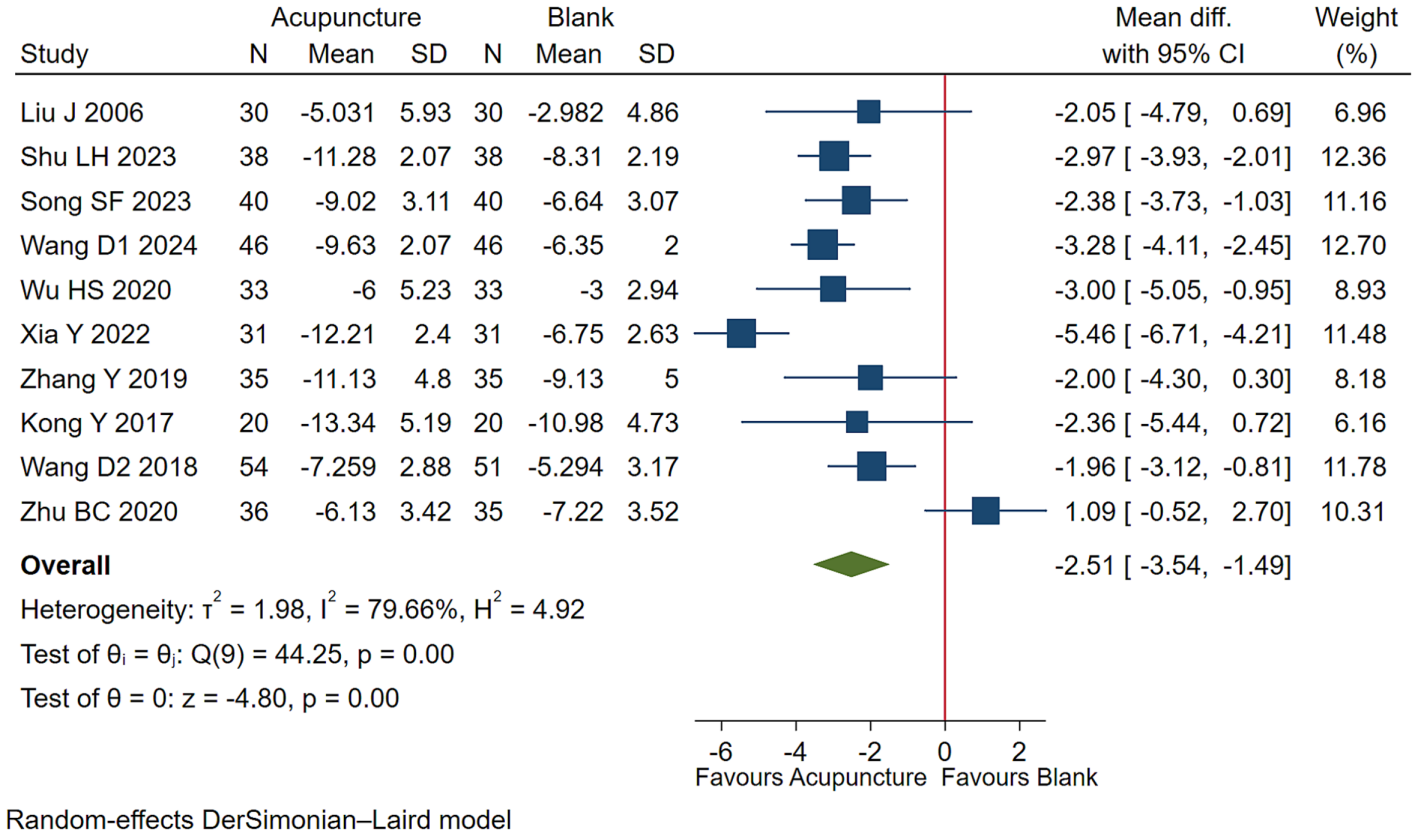
Acupuncture vs. blank control on basis of usual care evaluation of efficacy of motor tics.

Low-quality evidence (10 studies, 722 participants) suggested that acupuncture may lessen more motor tics symptoms in children with TS than blank treatment on basis of usual care [WMD −2.51, 95% CI (−3.54, −1.49), RD 0.31 (0.19, 0.41) (Figure 7; Table 3)] (38, 41–43, 45–47, 49–51).

**Table 3:** GRADE evidence quality rating of the clinical efficacy of acupuncture versus blank therapy for children with TS.

Comparison	No. of trials (No. of patients)	Follow-up days	Risk of bias	Inconsistency	Indirectness	Imprecision	Publication bias	Treatment association (95% CI)		Overall quality of evidence
Acupuncture vs. Blank	Motor Tic Score,0–25, lower is better									
	10 (722)	28 to 90	Serious^a^	Serious *I*^2^ = 79.66%	Not serious	Not serious	NA	WMD −2.51 (−3.54, −1.49)		Low
								RD 0.31 (0.19, 0.41)		
								Acupuncture 70.8%	Blank 29.1%	
	Vocal Tic Score, 0–25, lower is better									
	10 (722)	28 to 90	Serious^a^	Serious *I*^2^ = 81.05%	Not serious	Not serious	NA	WMD −2.56 (−3.66, −1.45)		Low
								RD 0.28 (0.15, 0.40)		
								Acupuncture 52.2%	Blank 24.7%	
	Impairment Score, 0–50, lower is better									
	2 (100)	30 to 90	Serious^a^	Not serious *I*^2^ = 0%	Not serious	Serious^b^	NA	WMD −2.91 (−4.64, −1.19)		Low^d^
								RD 0.13 (0.05, 0.23)		
								Acupuncture 27.3%	Blank 14.4%	
	Global Severity Score, 0–100, lower is better									
	14 (935)	28 to 90	Serious^a^	Not serious *I*^2^ = 68.07%	Not serious	Serious^c^	NA	WMD −5.57 (−7.47, −3.68)		Low
								RD 0.11 (0.06, 0.17)		
								Acupuncture 17%	Blank 6%	
	Adverse reactions									
	6 (411)	28 to84	Serious^a^	Not serious *I*^2^ = 31.3%	Not serious	Serious^b^	NA	RR 0.56, 95%CI (0.30,1.04)		Low

NA, not available.

a, high risk bias in blinding and randomization; b, small sample size; c, we rated down two levels for imprecision when the ratio of the upper to the lower limits of the CI is more than three for risk ratio based on GRADE guidance; d, Subgroup analyses were conducted.

#### 3.5.2 Vocal tic symptoms

**Figure 8:**
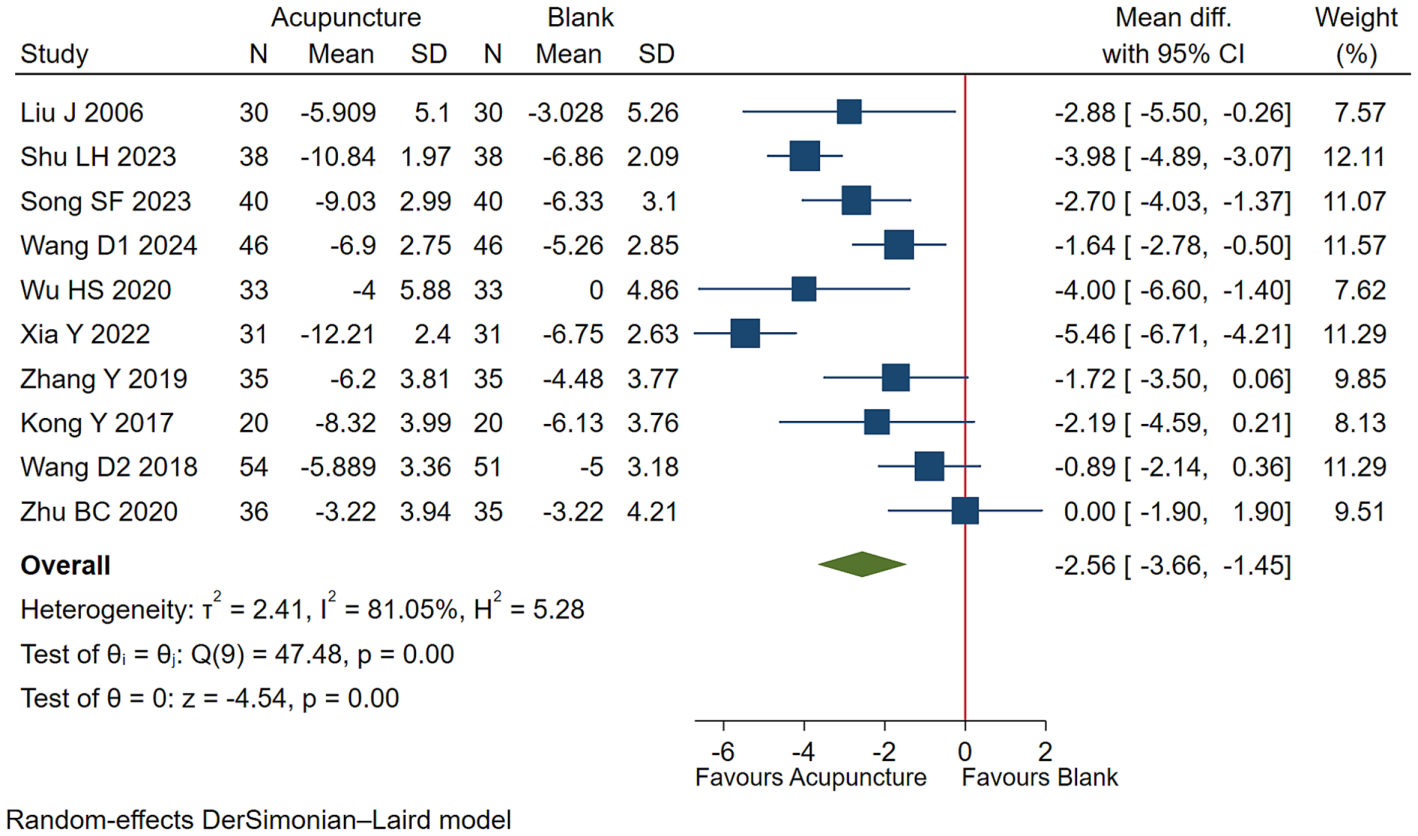
Acupuncture vs. blank control on basis of usual care evaluation of efficacy of vocal tics.

Low-quality evidence (10 studies, 722 participants) suggested that acupuncture may improve vocal tics symptoms in children with TS compared to blank treatment on basis of usual care [WMD −2.56, 95% CI (−3.66, −1.45), RD 0.28 (0.15, 0.40) (Figure 8; Table 3)] (38, 41–43, 45–47, 49–51).

#### 3.5.3 Functional impairment

**Figure 9:**
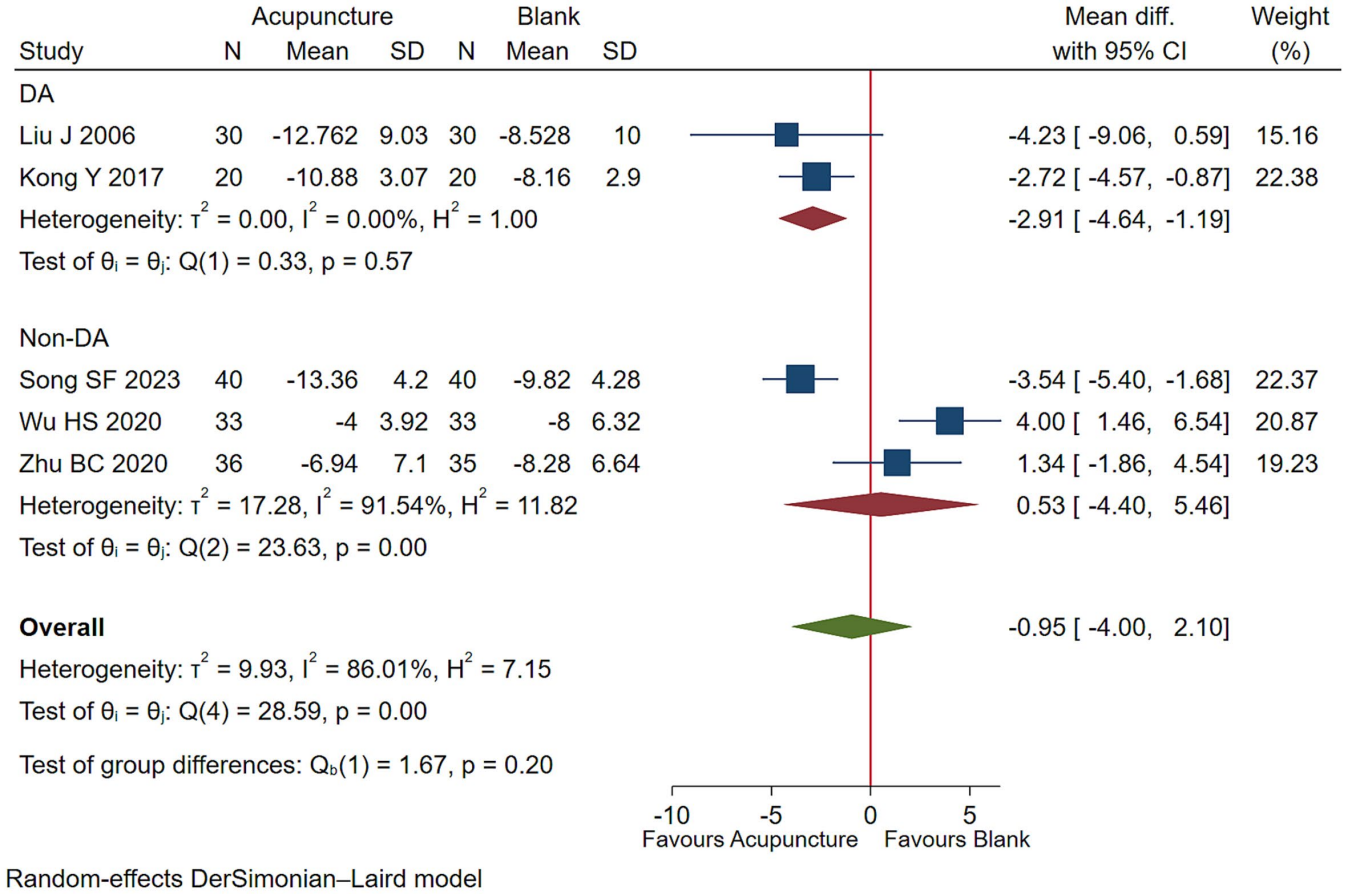
Acupuncture vs. blank control on basis of usual care evaluation of efficacy of functional improvement.

Low-quality evidence (2 studies, 100 participants) suggested that acupuncture with DA may lessen functioning impairment than DA [WMD −2.91, 95% CI (−4.64, −1.19), RD 0.13 (0.05, 0.23) (Figure 9; Table 3)] (38, 42, 45, 49, 51).

#### 3.5.4 Overall symptom severity

**Figure 10:**
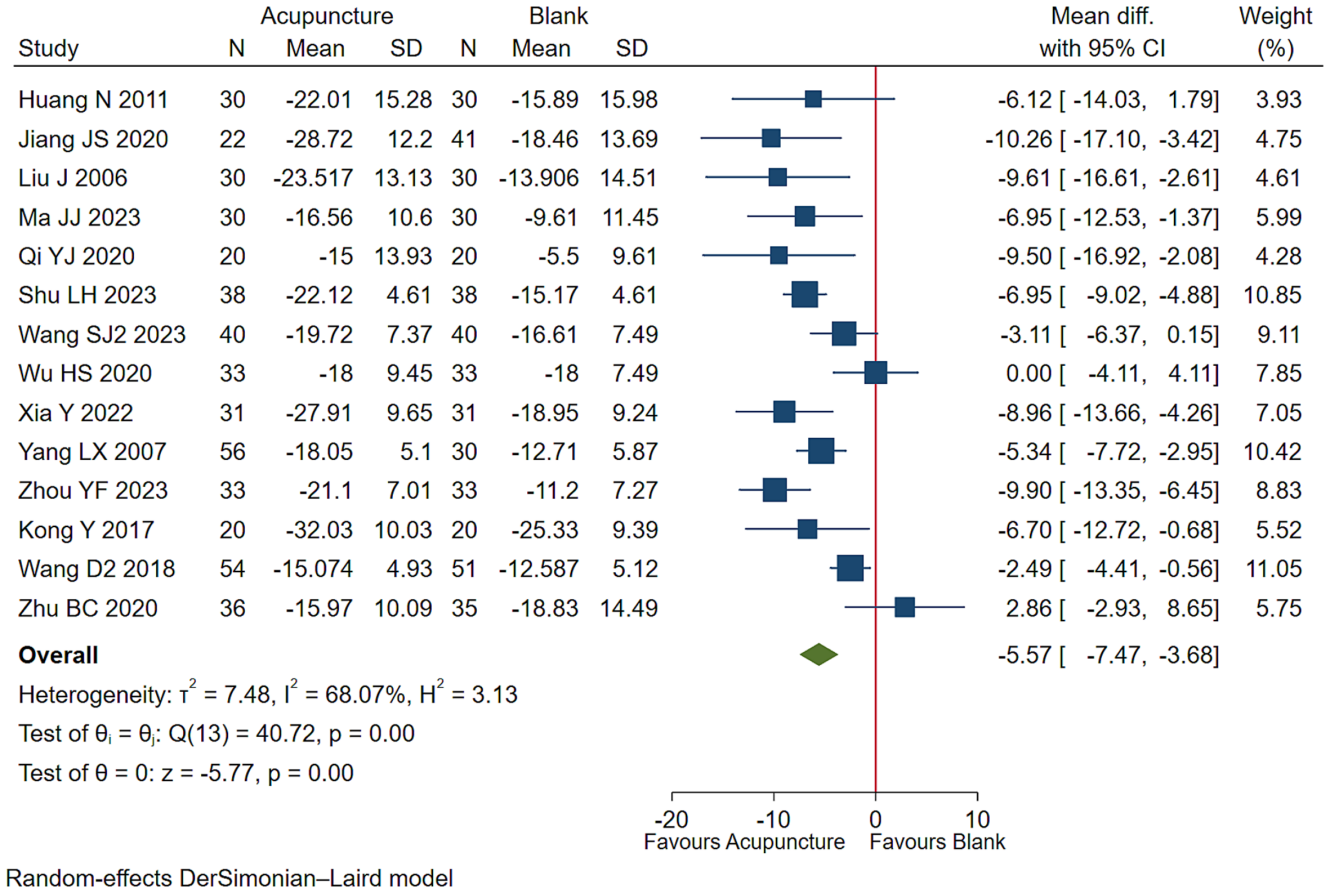
Acupuncture vs. blank control on basis of usual care evaluation of efficacy of overall symptom efficacy evaluation.

Low-quality evidence (14 studies, 935 participants) suggested that acupuncture may be superior in improving overall symptoms severity in children with TS than blank treatment [WMD −5.57, 95% CI (−7.47, −3.68), RD 0.11 (0.06, 0.17) (Figure 10; Table 3)] (32, 37–41, 44–46, 48–51, 53).

#### 3.5.5 Adverse reactions

**Figure 11:**
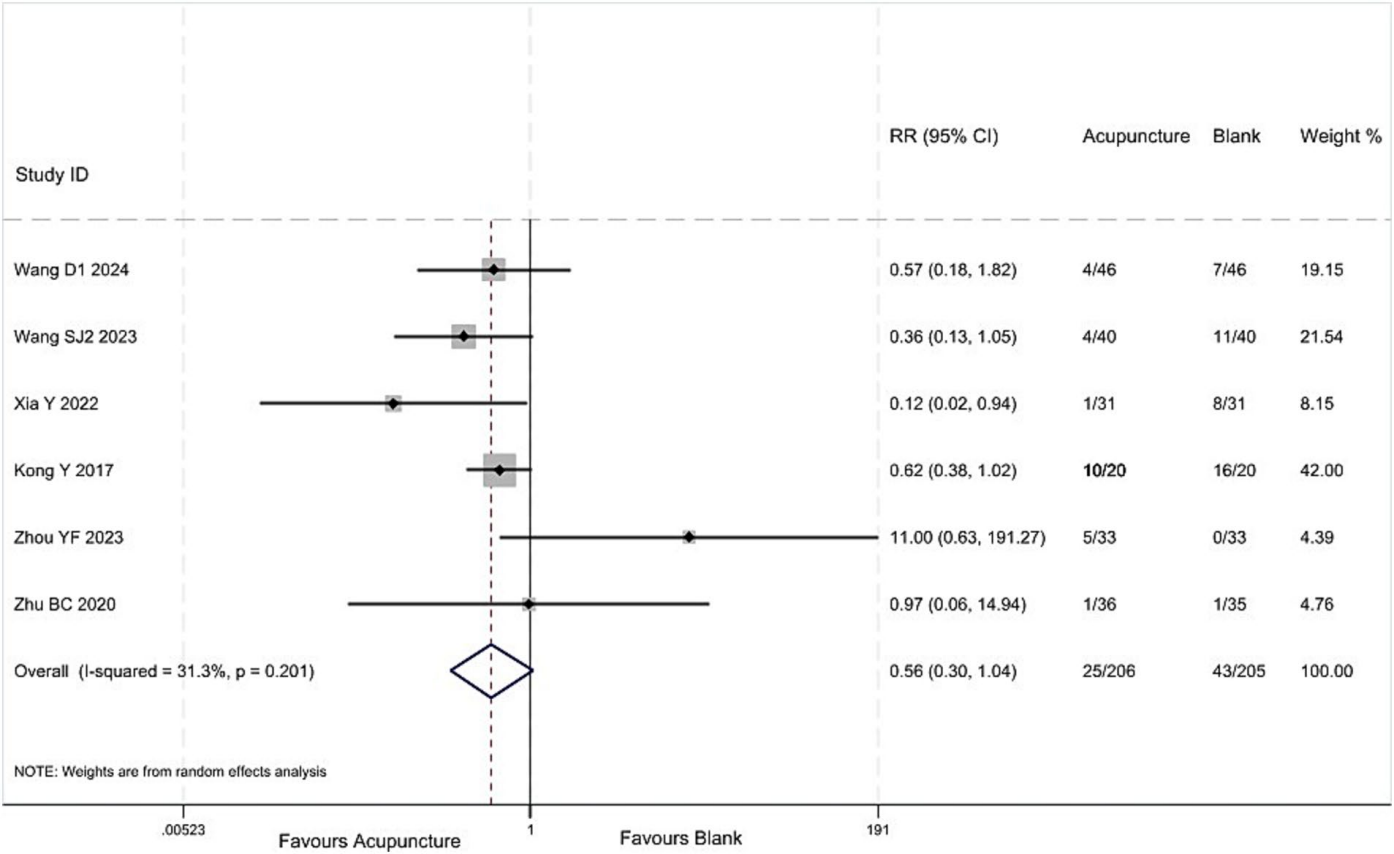
Acupuncture vs. blank control on basis of usual care analysis of the incidence of adverse reactions.

Low-quality evidence (6 studies, 411 participants) suggested that the incidence of adverse reactions in children with TS treated with acupuncture combined with other therapies is not significantly different from that in children treated with other therapies alone, [RR 0.56, 95%CI (0.30, 1.04), (Figure 11; Table 3)] (43, 44, 46, 48, 49, 51).

## 4 Discussion

### 4.1 Overall findings

Compared with pharmacotherapy, acupuncture may alleviate more motor tics symptoms with less adverse effects, but it may bring about slight improvement in vocal tics, functional impairments, and overall symptom severity in Chinese children with TS. Compared with blank treatment, acupuncture may alleviate more motor tics, vocal tics symptoms, functional impairments, and overall symptom severity in Chinese children with TS.

### 4.2 Relation of other studies

We identified eleven systematic reviews on acupuncture treatment for childhood tic disorders (11–21). After screening their included studies, we excluded those that enrolled patients with chronic or transient tic disorders, or that used ineligible comparators or outcomes (Supplementary Table 2).

The latest systematic review (21) included 26 studies, with a search cut-off date of October 2023. The results indicated that acupuncture is more effective than most existing treatments in alleviating motor and vocal tics in children with TS, while also reducing the incidence of adverse reactions. However, some outcome measures in the included studies only reported treatment efficacy rates, failing to clearly assess improvements in tic symptom-related scores. Additionally, the study did not include outcomes related to functional impairments in children with TS, focusing solely on motor and vocal tics. Finally, the study did not assess the certainty of the evidence, lacking a certain degree of professionalism.

This study included 18 RCTs not included in the latest review and excluded 12 studies included in the review, with specific reasons as follows: (1) Six studies had questionable inclusion criteria for children with TS, including patients with chronic tic disorder and transient tic disorder (54–59), (2) Two studies had acupuncture intervention durations of less than 28 days (60, 61), (3) Four studies did not report reasonable tic symptom scores (62–65). Our findings lead to a more cautious conclusion than the latest review and emphasize that the role of acupuncture in the treatment of TS in children requires further clinical research to confirm. (Supplementary Table 2).

### 4.3 Strengths and limitations

The strengths of this study include a comprehensive search for eligible RCTs and a focus on patient-reported outcome measures, with analysis conducted across three domains: motor tics, vocal tics, and functional impairment. We used the GRADE method to assess the certainty of the evidence and referenced the latest research results from the BMJ (66) and Cochrane Collaboration (10) for methodological exploration: first, we calculated the scores where the reduction rate in each YGTSS subscale reached at least 30%, defining this as the MCID. We then calculated the probability of achieving this value in the acupuncture group and the control group, respectively, and used the RD to display the between-group differences, thereby making the findings more accessible and clinically interpretable.

This study has limitations. Only three studies (22, 35, 36) adequately described random-sequence generation, allocation concealment, participant blinding, and implementation details. Additionally, due to the special nature of acupuncture procedures, it was not possible to blind patients or operators, resulting in a high risk of bias. Our review was based on the Chinese population, which limits its generalizability. Evidence from other populations is therefore needed for further analysis.

### 4.4 Implications

Chinese children with TS who receive acupuncture may experience greater relief of motor tics and fewer adverse effects compared with those on pharmacotherapy. Relative to no-treatment controls, these children also demonstrate larger reductions in vocal tics, functional impairment, and overall symptom severity. Low-certainty evidence underpins these conclusions on basis of Chinese population. Future trials should adopt rigorous methodological safeguards to minimize bias, and additional well-designed RCTs are required to clarify the efficacy of acupuncture for pediatric tic disorders.

## 5 Conclusion

Compared to pharmacotherapy, acupuncture may relieve more motor tics symptoms with less adverse effects, but it may slightly improve vocal tics, functional impairments, and overall symptom severity in Chinese children with TS. Compared to blank treatment, acupuncture may alleviate more motor tics, vocal tics symptoms, functional impairments, and overall symptom severity in Chinese children with TS. All results are supported by low-quality evidence.

## Data Availability

The original contributions presented in the study are included in the article/Supplementary material, further inquiries can be directed to the corresponding author.
