# Supplementary material for: The effectiveness of acupuncture in the treatment of Tourette syndrome in Chinese children: a systematic review and meta-analysis
Source: Front Public Health. 2025 Oct 2;13:1677592. doi: 10.3389/fpubh.2025.1677592 (PMC12528205; doi:10.3389/fpubh.2025.1677592)
Supplement: Supplementary file 1 [file Table_1.DOCX]

SUPPLEMENTARY TABLE 1 Database search terms and search results

| **Database** | **Search** | **Results** |
| --- | --- | --- |
| **CNKI** | Theme=(针刺 + 针刺治疗 + 针刺方法 + 针刺疗法 + 联合针刺 + 取穴针刺 + 针灸 + 头针 + 针 + 针灸 + 电针 + 手针 + 假针刺 + 联合针刺 + 电针针刺 + 针法) AND (儿童抽动症 + 儿童抽动秽语综合征 + 儿童抽动障碍 + '儿童抽动-秽语综合征' + 儿童抽动障碍症 + 多发性抽动 + 图雷特 + 抽动症 + 抽动障碍 + 慢性抽动障碍 + 抽动秽语 + Tourette + 多发性抽动) | **559** |
| **Wan Fang** | Theme =(针刺 OR 针刺治疗 OR 针刺方法 OR 针刺疗法 OR 联合针刺 OR 取穴针刺 OR 针灸 OR 头针 OR 针 OR 针 OR 针灸 OR 电针 OR 手针 OR 假针刺 OR 针法) AND (儿童抽动症 OR 儿童抽动秽语综合征 OR 儿童抽动障碍 OR '儿童抽动-秽语综合征' OR 儿童抽动障碍症 OR 多发性抽动 OR 图雷特 OR 抽动症 OR 抽动障碍 OR Tourette) | **723** |
| **VIP** | Theme =(针刺 OR 针刺治疗 OR 针刺方法 OR 针刺疗法 OR 联合针刺 OR 取穴针刺 OR 针灸 OR 头针 OR 针 OR 针灸 OR 电针 OR 手针 OR 假针刺 OR 针法)  AND (儿童抽动症 OR 儿童抽动秽语综合征 OR 儿童抽动障碍 OR 多发性抽动 OR 图雷特 OR 抽动症 OR 抽动障碍 OR 多发性抽动) | **339** |
| **Cochrane** | #1 'Tourette syndrome' OR 'Tic disorder' OR 'Gilles de la Tourette's Disease' OR 'Tourettes Disorder' OR 'Tics' OR 'Gestural Tic' OR 'Childhood Tic Disorder' OR 'Motor Tic' OR 'Transient Tic' OR 'Combined Multiple Motor and Vocal Tic Disorder'  #2 'de la Tourette disease' OR 'Gilles de la Tourette disease' OR 'Gilles de la Tourette’s syndrome' OR 'Gilles de la Tourette’s syndrome' OR 'Gilles de Tourette syndrome' OR 'Tourette disease' OR 'Tourette syndrome' OR 'Tourette’s syndrome' OR 'Tourette’s syndrome' OR 'Gilles de la Tourette syndrome' #3 'facial twitching' OR 'habit spasm' OR 'nervous tic' OR 'nervous twitch' OR 'spasm, habit' OR 'tic disorder' OR 'tic disorders' OR 'tics' OR 'tic' #4 #1 OR #2 OR #3 #5'pharmacoacupuncture treatment' OR 'treatment, pharmacoacupuncture' OR 'pharmacoacupuncture therapy' OR 'therapy, pharmacoacupuncture' OR 'acupotomy' OR 'acupotomies' OR 'acupuncture point' OR 'point, acupuncture' OR 'points, acupuncture' OR 'acupoints' OR 'acupoint' OR 'analgesia, acupuncture' OR 'acupuncture anesthesia' OR 'anesthesia, acupuncture' OR ‘needle’ #6'acupuncture' OR 'acupuncture treatment' OR 'pharmacopuncture' OR 'acupuncture treatments' OR 'treatment, acupuncture' OR 'therapy, acupuncture' OR 'pharmacoacupuncture treatment' OR 'treatment, pharmacoacupuncture' OR 'pharmacoacupuncture therapy' OR 'therapy, pharmacoacupuncture' OR 'acupotomy' OR 'acupotomies' OR 'acupuncture point' OR 'point, acupuncture' OR 'points, acupuncture' OR 'acupoints' OR 'acupoint' OR 'analgesia, acupuncture' OR 'acupuncture anesthesia' OR 'anesthesia, acupuncture' #7 #5 or #6 #8 #7 AND #4 | **62** |
| **Embase** | #1'gilles de la tourette syndrome'/exp OR 'gilles de la tourette syndrome' OR (gilles AND de AND ('la'/exp OR la) AND tourette AND ('syndrome'/exp OR syndrome)) #2 'de la tourette disease' OR 'gilles de la tourette disease' OR 'gilles de la tourette`s syndrome' OR 'gilles de la tourettes syndrome' OR 'gilles de tourette syndrome' OR 'tourette disease' OR 'tourette syndrome' OR 'tourette`s syndrome' OR 'tourettes syndrome' OR 'gilles de la tourette syndrome' #3 'facial twitching' OR 'habit spasm' OR 'nervous tic' OR 'nervous twitch' OR 'spasm, habit' OR 'tic disorder' OR 'tic disorders' OR 'tics' OR 'tic' #4---#1 OR #2 OR #3 #5(tic AND disorder AND motor OR tic) AND (disorders OR vocal) AND tic AND disorder OR chronic) AND motor OR vocal) AND tic AND disorder #6 #4 OR #5 #7 'acupuncture' OR 'acupuncture treatment' OR 'pharmacopuncture' OR 'acupuncture treatments' OR 'treatment, acupuncture' OR 'therapy, acupuncture' OR 'pharmacoacupuncture treatment' OR 'treatment, pharmacoacupuncture' OR 'pharmacoacupuncture therapy' OR 'therapy, pharmacoacupuncture' OR 'acupotomy' OR 'acupotomies' OR 'acupuncturepoint' OR 'point, acupuncture' OR 'points, acupuncture' OR 'acupoints' OR 'acupoint' OR 'analgesia, acupuncture' OR 'acupuncture anesthesia' OR 'anesthesia, acupuncture' #8 needle #9 #7 OR #8 #10 #6 AND #9 | **297** |
| **PubMed** | #1 ((((((((((((((((tics [Mesh Terms]) OR (Tic, Vocal[Title/Abstract])) OR (Tics, Vocal[Title/Abstract])) OR (Vocal Tic[Title/Abstract])) OR (Vocal Tics[Title/Abstract])) OR (Tic, Transient[Title/Abstract])) OR (Tics, Transient[Title/Abstract])) OR (Transient Tic[Title/Abstract])) OR (Transient Tics[Title/Abstract])) OR (Tic, Gestural[Title/Abstract])) OR (Gestural Tic[Title/Abstract])) OR (Gestural Tics[Title/Abstract])) OR (Tics, Gestural[Title/Abstract])) OR (Tic, Motor[Title/Abstract])) OR (Motor Tic[Title/Abstract])) OR (Motor Tics[Title/Abstract])) OR (Tics, Motor[Title/Abstract]) #2 ((((((((((((((tourette syndrome[Mesh Terms]) OR (Tic Disorder, Combined Vocal[Title/Abstract] AND Multiple Motor[Title/Abstract])) OR (Tourette Disease[Title/Abstract])) OR (Tourette Disorder[Title/Abstract])) OR (Tourette's Disease[Title/Abstract])) OR (Tourettes Disease[Title/Abstract])) OR (Tourette's Disorder[Title/Abstract])) OR (Tourettes Disorder[Title/Abstract])) OR (Chronic Motor[Title/Abstract] AND Vocal Tic Disorder[Title/Abstract])) OR (Gilles De La Tourette's Syndrome[Title/Abstract])) OR (Gilles de la Tourette Disorder[Title/Abstract])) OR (Combined Multiple Motor[Title/Abstract] AND Vocal Tic Disorder[Title/Abstract])) OR (Combined Vocal[Title/Abstract] AND Multiple Motor Tic Disorder[Title/Abstract])) OR (Gilles de la Tourette Syndrome[Title/Abstract])) OR (Gilles de la Tourette's Disease[Title/Abstract]) #3 ((((((((((((((((((((((((((Tic disorders[Mesh Terms]) OR (Tic Disorder[Title/Abstract])) OR (Chronic Motor[Title/Abstract] OR Vocal Tic Disorder[Title/Abstract])) OR (Tic Disorder, Chronic Motor[Title/Abstract] OR Vocal[Title/Abstract])) OR (Motor[Title/Abstract] OR Vocal Tic Disorder, Chronic[Title/Abstract])) OR (Transient Tic Disorder[Title/Abstract])) OR (Tic Disorders, Transient[Title/Abstract])) OR (Transient Tic Disorders[Title/Abstract])) OR (Tic Disorder, Transient[Title/Abstract])) OR (Post-Traumatic Tic Disorder[Title/Abstract])) OR (Post Traumatic Tic Disorder[Title/Abstract])) OR (Post-Traumatic Tic Disorders[Title/Abstract])) OR (Tic Disorders, Post-Traumatic[Title/Abstract])) OR (Tic Disorder, Post-Traumatic[Title/Abstract])) OR (Tic Disorder, Post Traumatic[Title/Abstract])) OR (Tic Disorders, Vocal[Title/Abstract])) OR (Tic Disorder, Vocal[Title/Abstract])) OR (Vocal Tic Disorder[Title/Abstract])) OR (Vocal Tic Disorders[Title/Abstract])) OR (Childhood Tic Disorders[Title/Abstract])) OR (Childhood Tic Disorder[Title/Abstract])) OR (Tic Disorder, Childhood[Title/Abstract])) OR (Tic Disorders, Childhood[Title/Abstract])) OR (Motor Tic Disorders[Title/Abstract])) OR (Motor Tic Disorder[Title/Abstract])) OR (Tic Disorder, Motor[Title/Abstract])) OR (Tic Disorders, Motor[Title/Abstract]) #4 #1 OR #2 OR #3 #5 (((((((((((((((((((acupuncture[MeSH Terms]) OR (Acupuncture Treatment[Title/Abstract])) OR (Pharmacopuncture[Title/Abstract])) OR (Acupuncture Treatments[Title/Abstract])) OR (Treatment, Acupuncture[Title/Abstract])) OR (Therapy, Acupuncture[Title/Abstract])) OR (Pharmacoacupuncture Treatment[Title/Abstract])) OR (Treatment, Pharmacoacupuncture[Title/Abstract])) OR (Pharmacoacupuncture Therapy[Title/Abstract])) OR (Therapy, Pharmacoacupuncture[Title/Abstract])) OR (Acupotomy[Title/Abstract])) OR (Acupotomies[Title/Abstract])) OR (Acupuncture Point[Title/Abstract])) OR (Point, Acupuncture[Title/Abstract])) OR (Points, Acupuncture[Title/Abstract])) OR (Acupoints[Title/Abstract])) OR (Acupoint[Title/Abstract])) OR (Analgesia, Acupuncture[Title/Abstract])) OR (Acupuncture Anesthesia[Title/Abstract])) OR (Anesthesia, Acupuncture[Title/Abstract]) #6 #4 AND #5 | **1198** |
