# Supplementary material for: The effectiveness of acupuncture in the treatment of Tourette syndrome in Chinese children: a systematic review and meta-analysis
Source: Front Public Health. 2025 Oct 2;13:1677592. doi: 10.3389/fpubh.2025.1677592 (PMC12528205; doi:10.3389/fpubh.2025.1677592)
Supplement: Supplementary file 2 [file Table_2.DOCX]

SUPPLEMENTARY TABLE 2. The reasons to exclude included studies of previous systematic reviews

| **Reference ID** | **RCT** | **Reasons to exclude** |
| --- | --- | --- |
| 1 | Shan YH 2001 | ineligible outcome |
| 2 | Jin MZ 1998 | ineligible outcome |
| 3 | Zhang YH 2002 | ineligible outcome |
| 4 | Guo WH 2004 | ineligible outcome |
| 5 | Chi X 2004 | ineligible intervention |
| 6 | Zhang H 2005 | ineligible intervention |
| 7 | Mao HR 20005 | ineligible intervention |
| 8 | Sun DW 2005 | ineligible intervention |
| 9 | Xu N 2005 | ineligible outcome |
| 10 | Ma S 2006 | ineligible outcome |
| 11 | Du GS 2007 | ineligible outcome |
| 12 | Ge QY 2008 | ineligible outcome |
| 13 | Xu SF 2009 | duplicate literature |
| 14 | Zhang ZR 2009 | ineligible intervention |
| 15 | Jin YA 2010 | ineligible outcome |
| 16 | Liu HM 2010 | ineligible intervention |
| 17 | Xiang SJ 2010 | ineligible population |
| 18 | He LZ 2012 | ineligible intervention |
| 19 | Sun YZ 2014 | ineligible outcome |
| 20 | Jiang LF 2014 | ineligible intervention |
| 21 | Guo YX 2014 | ineligible outcome |
| 22 | Zhang XJ 2015 | ineligible outcome |
| 23 | Tang Y 2015 | ineligible intervention |
| 24 | Jiao JM 2016 | ineligible outcome |
| 25 | Chen YL 2016 | ineligible population |
| 26 | Xu CM 2017 | ineligible population |
| 27 | Xing XJ 2017 | ineligible intervention |
| 28 | Qian X 2017 | ineligible population |
| 29 | Hu TH 2018 | ineligible outcome |
| 30 | Yang N 2018 | ineligible intervention |
| 31 | Yan L 2019 | ineligible intervention |
| 32 | Shen LP 2019 | ineligible population |
| 33 | Li Y 2021 | ineligible intervention |
| 34 | Ye DL 2013 | ineligible intervention |
| 35 | Tu HS 2019 | ineligible outcome |
| 36 | Miao QX 2019 | ineligible outcome |
| 37 | Zhao RG 2017 | ineligible outcome |
| 38 | Kou SJ 2003 | ineligible intervention |
| 39 | Peng L 2019 | ineligible intervention |
| 40 | Li YR 2016 | ineligible population |
| 41 | Yang YM 2016 | ineligible intervention |
| 42 | Sun LX 2017 | ineligible intervention |
| 43 | Fan YN 2017 | ineligible intervention |
| 44 | Tian ZW 2018 | ineligible population |
| 45 | Guo MR 2021 | ineligible population |
| 46 | Huang LZ 2019 | ineligible population |
| 47 | Luo W 2021 | ineligible population |
| 48 | Xuan YJ 2019 | ineligible population |
| 49 | Huang Y 2021 | ineligible population |
| 50 | Huang J 2022 | ineligible intervention |
| 51 | Fang JX 2013 | ineligible outcome |
| 52 | Shi XH 2021 | ineligible outcome |
| 53 | Wu HS 2021 | ineligible outcome |
| 54 | Liu YZ 2009 | duplicate literature |
