# Supplementary material for: The effectiveness of acupuncture in the treatment of Tourette syndrome in Chinese children: a systematic review and meta-analysis
Source: Front Public Health. 2025 Oct 2;13:1677592. doi: 10.3389/fpubh.2025.1677592 (PMC12528205; doi:10.3389/fpubh.2025.1677592)
Supplement: Supplementary file 3 [file Table_3.DOCX]

SNPPLEMENT TABLE 3. Risk bias of included studies

| **Study ID** | **Sequence generation** | **Allocation concealment** | **Blinding of patients** | **Blinding of health care providers** | **Blinding of data collectors** | **Blinding of outcome assessors** | **Blinding of data analyst** | **Loss to follow-up (%)** |
| --- | --- | --- | --- | --- | --- | --- | --- | --- |
| Le W 2022 | Low | Low^a^ | High | High | Low ^a^ | Low ^a^ | Low ^a^ | 0 |
| Li YC 2016 | High ^a^ | High | High | High | High | High | High | 0 |
| Liu H 2016 | Low | High | High | High | High | High | High | 0 |
| Liu L 2010 | High ^b^ | High | High | High | High | High | High | 0 |
| Mu JP 2009 | Low | High | High | High | High | High | High | 0 |
| Ni W 2016 | Low | High | High | High | High | High | High | 5% |
| Song Y 2022 | Low | High | High | High | High | High | High | 0 |
| Sun YZ 2018 | Low | High | High | High | High | High | High | 0 |
| Wang SJ1 2020 | High ^b^ | High | High | High | High | High | High | 0 |
| Xu SF 2009 | High ^a^ | High | High | High | High | High | High | 0 |
| Huang N 2011 | Low | High | High | High | High | High | High | 5% |
| Yin ZQ 2024 | Low | High | High | High | High | High | High | 0 |
| Zhang ML 2024 | Low | High | High | High | High | High | High | 0 |
| Hu CY 2022 | High ^a^ | High | High | High | Low ^a^ | Low ^a^ | Low ^a^ | 0 |
| Dong ZW 2022 | Low | High | High | High | Low ^a^ | Low ^a^ | Low ^a^ | 0 |
| Jiang JS 2020 | High ^c^ | High | High | High | High | High | High | 10% |
| Liu J 2006 | High ^a^ | High | High | High | High | High | High | 0 |
| Ma JJ 2023 | Low | High | High | High | High | High | High | 0 |
| Qi YJ 2020 | Low | High | High | High | High | High | High | 0 |
| Shu LH 2023 | Low | High | High | High | High | High | High | 0 |
| Song SF 2023 | Low | High | High | High | High | High | High | 0 |
| Wang D1 2024 | Low | High | High | High | High | High | High | 0 |
| Wang SJ2 2023 | Low | High | High | High | High | High | High | 0 |
| Wu HS 2020 | Low | Low ^a^ | High | High | High | High | High | 0 |
| Xia Y 2022 | Low | High | High | High | High | High | High | 0 |
| Yang LX 2007 | Low | High | High | High | High | High | High | 4.4% |
| Zhang Y 2019 | Low | High | High | High | High | High | High | 0 |
| Zhou YF 2023 | Low | Low ^a^ | High | High | High | High | High | 5.7% |
| Kong Y 2017 | Low | High | High | High | High | High | High | 0 |
| Wang D2 2018 | Low | High | High | High | High | High | High | 0 |
| Zhu BC 2020 | Low | High | High | High | High | High | High | 11.25% |
| Yu PB 2019 | Low | High | High | High | High | High | High | 0 |

Sequence generation Low: Use random number tables, random number tables combined with random sequences generated based on the order of visits

Adequate randomization sequence generation High ^a^: Only describe random terms

Adequate randomization sequence generation High ^b^: Randomly based on the order of visits

Adequate randomization sequence generation High ^c^: Randomly grouped based on order of visit and whether acupuncture was accepted

Allocation concealment High: Allocated according to random number tables

Allocation concealment Low^a^: Hide the serial numbers in opaque, sealed envelopes and allocate them randomly.

Blinding of patients、Blinding of health care providers: Due to the special characteristics of acupuncture, it is impossible to blind patients and operators

Blinding of data collectors、Blinding of outcome assessors、Blinding of data analyst High：Not described in the study

Blinding of data collectors、Blinding of outcome assessors、Blinding of data analyst Low^a^：The study mentions that it was conducted by a third party

Loss to follow-up (%): The study mentioned that a dropout rate > 20% was considered high risk
